# Glaucomatocyclitic Crises May Occur in Patients with Narrow or Closed Angles

**DOI:** 10.1155/2017/4074912

**Published:** 2017-11-19

**Authors:** Pei-Kang Liu, Han-Yi Tseng, Min-Yu Huang, Kwou-Yeung Wu

**Affiliations:** ^1^Department of Ophthalmology, Kaohsiung Medical University Hospital, Kaohsiung Medical University, Kaohsiung, Taiwan; ^2^Department of Ophthalmology, Yuan's General Hospital, Kaohsiung, Taiwan; ^3^Department of Ophthalmology, Kaohsiung Municipal Hsiao-Kang Hospital, Kaohsiung Medical University, Kaohsiung, Taiwan; ^4^Department of Ophthalmology, Kaohsiung Municipal Ta-Tung Hospital, Kaohsiung Medical University, Kaohsiung, Taiwan; ^5^Department of Ophthalmology, School of Medicine, Kaohsiung Medical University, Kaohsiung, Taiwan

## Abstract

**Purpose:**

To report cases of glaucomatocyclitic crises and discuss the possibility of occurrence in patients with narrow or closed angles.

**Background:**

The prevalence of angle closure is much higher among Asians than among the Western population. Currently, there is no evidence for a direct relationship between the etiology and angle structure.

**Design:**

A retrospective and observational case series.

**Methods:**

We retrospectively collected data from nine adult patients (three males and six females) who were diagnosed with a glaucomatocyclitic crisis and a shallow anterior chamber over a 21-year period, from 1995 to 2016, at the Kaohsiung Medical University Hospital. A narrow angle was defined as a grade less than the Shaffer system grade II. Ophthalmic examinations, including anterior segment biomicroscopy, direct ophthalmoscopy, intraocular pressure measurements, anterior chamber reaction, visual field tests, and the grade of the anterior chamber angle according to the Shaffer system, were reviewed.

**Results:**

These patients experienced at least one typical unilateral ocular hypertensive episode that fulfilled the criteria of a glaucomatocyclitic crisis without the angle feature. All patients had gonioscopically narrow or closed angles with or without peripheral anterior synechiae.

**Conclusions:**

The coexistence of narrow or closed angles and a glaucomatocyclitic crisis is possible, especially in patients of Asian descent. In patients with shallow anterior chambers, a glaucomatocyclitic crisis may be a cause of acute glaucoma episodes.

## 1. Introduction

Glaucomatocyclitic crisis, also known as the Posner-Schlossman syndrome (PSS), is a self-limiting, rare disease that was first described by Posner and Schlossman in 1948 [1]. This relatively uncommon disease consists of unilateral recurrent attacks of increased intraocular pressure (IIOP) associated with mild anterior uveitis, a lack of visual field defects, normal optic discs, and an open angle [1–3]. PSS is classified as a secondary inflammatory glaucoma because uveitis always accompanies the disease [2, 4]. The mild nature of the inflammation at the presentation of the first attack and the rarity of clinical symptoms may cause the disease to be overlooked. Mild nongranulomatous cyclitis may follow acute IIOP by several days; therefore, the etiology of an IIOP episode may be initially unclear. Fine, discrete, round, white keratic precipitates (KP) are characteristic of the disease and may be found on the trabecular meshwork on gonioscopy. Spontaneous resolution of KP may occur within a few weeks [2]. In contrast to the common presentations of severe pain and nausea in acute angle-closure glaucoma episodes, PSS attacks involve only mild discomfort. The affected eye may show mild or no corneal edema with minimal or even no congestion despite marked IIOP. The common symptoms include ocular fullness sensation, blurred vision, or halos. Typically, peripheral anterior synechiae (PAS) or posterior synechiae are uncommon in this disease despite the presence of an anterior chamber reaction [1]. PSS tends to affect young to middle-aged adults between the second and fifth decades [5]. The mean annual incidence and prevalence rates are 0.4 and 1.9 per 100,000 individuals, respectively [6].

The etiology of this disease remains unclear, and potential contributing factors include cytomegalovirus infection [7, 8], autonomic defects/dysregulation [1, 9, 10], autoimmune disease [11–13], herpes simplex virus infection [14, 15], *Helicobacter pylori* infection [16], and insufficient ocular blood supply due to peripheral vascular endotheliopathy [17]. The depth of the anterior chamber may decrease with age, especially in Asians [18]. Clinically, we observed that some patients who were diagnosed with typical PSS with a wide, open angle and suffered from recurrent attacks also had a narrow or closed angle due to aging. The possible etiology mentioned above does not directly relate to angle structure. Therefore, we hypothesized that PSS may occur in patients with a narrow or closed angle.

In this article, we present several cases of glaucomatocyclitic crises with narrow or closed angles.

## 2. Materials and Methods

The study consisted of consecutive, retrospective, observational case reviews. The described research adhered to the principles of the Declaration of Helsinki, 1964. We retrospectively collected data from patients who were diagnosed with glaucomatocyclitic crises and a shallow anterior chamber at the Kaohsiung Medical University Hospital over a twenty-one-year period from 1995 to 2016. All patients underwent ophthalmic examinations, including anterior segment biomicroscopy, direct ophthalmoscopy, and intraocular pressure (IOP) measurements. IOP was measured by the standard Goldmann applanation tonometry. Visual field tests were performed after the alleviation of IIOP. The angle was examined with an indirect single mirror goniolens and graded by the Shaffer system. A narrow angle was defined as a grade of less than the Shaffer system grade II.

## 3. Results

**Figure 1:**
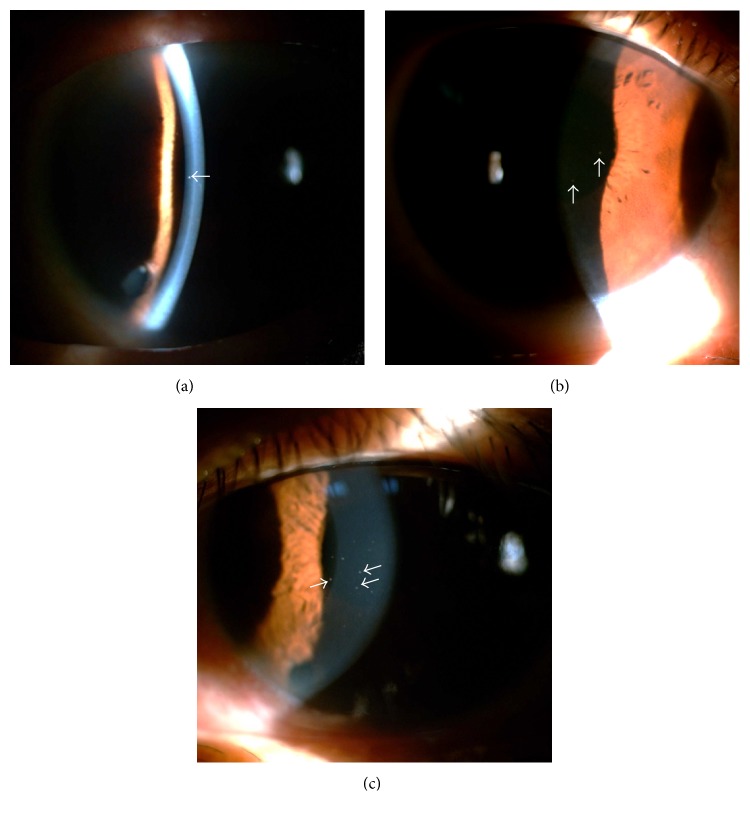
Small, discrete, round, well-defined, white keratic precipitates on the cornea. Red arrows indicate fine, discrete, round, white keratic precipitates on the endothelium of case 2 (a), case 8 (b), and case 9 (c).

**Figure 2:**
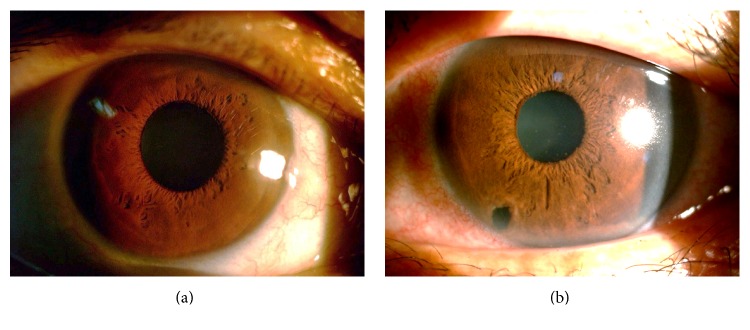
Relatively clear corneas were noted when glaucomatocyclitic crises occurred. The images of case 8 ((a) IOP = 50 mmHg) and case 9 ((b) IOP = 39 mmHg) revealed relatively minimal corneal edema when episodes of acute increased intraocular pressure occurred. IOP = intraocular pressure.

**Figure 3:**
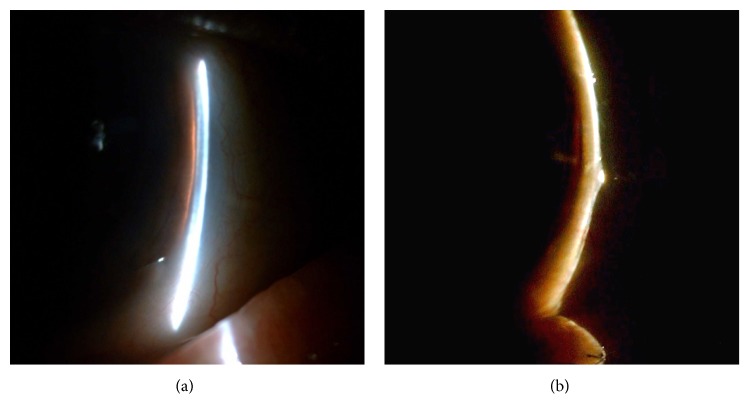
A narrow angle was identified according to the Van Herick technique. Shallow anterior chamber depth was identified through an anterior segment biomicroscopy examination using the Van Herick technique in case 2 (a) and case 8 (b).

**Figure 4:**
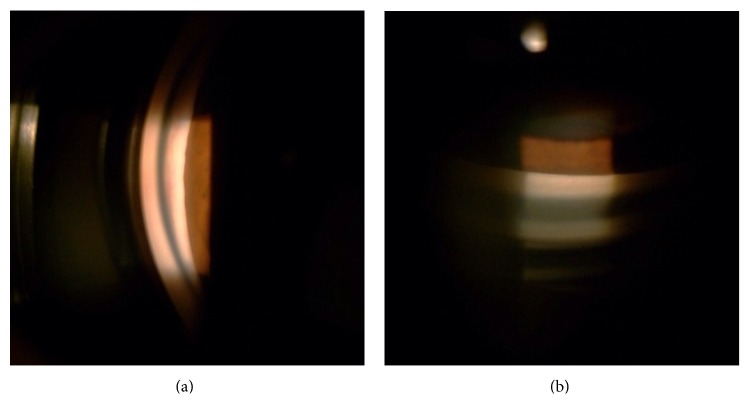
Example of angle structure images. The Shaffer system grade I angle structure was noted on an indirect single mirror goniolens in case 8 (a, b) before receiving laser peripheral iridotomy.

Nine patients (three males and six females) were enrolled in the study (Table 1). These patients had at least one typical unilateral ocular hypertensive episode that fulfilled the criteria of glaucomatocyclitic crisis (Table 2, Figures 1 and 2) without the angle feature. All patients demonstrated mild anterior uveitis with fine white KP when the recurrent glaucomatocyclitic-like episodes happened. There was no sign of uveitis between the episodes. They all had a gonioscopically narrow or closed angle with or without PAS (Figures 3 and 4). No atrophic iris change was detected in these cases. Six patients were first diagnosed with PSS at the time of the ocular hypertensive episodes, but narrow angles were subsequently found gonioscopically by a glaucoma specialist (cases 1, 2, 4, 5, 6, and 8). Three patients had received preventive laser iridotomies for occludable angles before the ocular hypertensive episodes occurred, but glaucomatocyclitic-like attacks occurred afterwards (cases 3, 7, and 9). All patients received laser iridotomies on both eyes after narrow angles were identified, before or after the glaucomatocyclitic-like episodes. The uveitic work up including human leukocyte antigen (HLA) B27 and antinuclear antibodies (ANA) was collected in cases 1, 2, and 8. All the results were negative. All patients were not on long-term glaucoma medications before the first episode. During follow-up, three patients (cases 1, 2, and 3) needed long-term glaucoma medications for the control of IOP due to subsequent glaucoma and progression of visual field defect.

## 4. Discussion

Glaucomatocyclitic crisis was first reported and described by Posner and Schlossman decades ago [1, 3]. In their report, a series of 9 patients with ocular hypertension shared the same characteristics (Table 2). PSS typically occurs in individuals aged 20–50 years. However, an atypical episode in a 13-year-old adolescent [19] as well as attacks in adults older than 60 years has been reported. PSS is generally considered a benign disease. Neither development of PAS nor posterior synechiae is a classical symptom of PSS [1]. IIOP is typically disproportionate to the inflammatory reaction of the anterior chamber and precedes the reaction by several days. Fine, discrete, round, white KP may be found on the trabecular meshwork.

Most PSS patients adequately treated for acute episodes of IOP elevation completely recover without long-term sequelae, while a portion of patients may develop glaucomatous optic nerve damage and various visual field defects after repeated events [20]. However, treatment during remission is not necessary because the frequency of further attacks is not influenced by unremitting treatment. In 1973, Kass et al. established a positive association between glaucomatocyclitic crisis and primary open angle glaucoma (POAG) in an observation of eleven patients. They reported up to 45% concomitance between PSS and POAG [21]. Jap et al. also found that 26.4% of PSS patients developed glaucomatous optic nerve damage [20].

A number of cases have been reported with some deviation from the primary descriptions by Posner and Schlossman, such as a bilateral simultaneous presentation [22, 23], prominent iris processes, anterior displacement of Schwalbe's line, or a fine membrane over the trabecular meshwork [24, 25], although the presence of angle abnormalities is not considered a typical diagnostic characteristic by some experts. However, no previous studies have challenged the open angle concept.

In 2007, Green reported on a 47-year-old Vietnamese male initially diagnosed with acute angle-closure glaucoma and subsequently with PSS [5]. After receiving laser iridotomy in the affected eye, the patient suffered from several episodes of IIOP with mild conjunctival hyperemia, a few fine KP, and mild cyclitis with wide open angles (Schaffer grades 3 to 4) on gonioscopy. However, the diagnosis of PSS was challenged by the author because sectoral iris atrophy was identified in this case, and the Fuchs heterochromic iridocyclitis may have been an alternative diagnosis.

The definite causes of PSS remain unclear. To date, no evidence has shown a direct relationship between the etiology and angle structure. The prevalence of angle closure is much higher among Asians than among the Western population, and a more rapid decline in angle width measurements is apparent with age in the general population [18]. Asians also have a higher prevalence of primary angle-closure glaucoma [26–28], and the risk factors include Asian descent [29–31], older age [29, 32], and shorter axial length [33]. Therefore, it is reasonable that glaucomatocyclitic episodes occur in Asian patients with narrow angles.

However, presentation with a narrow or closed angle inevitably raises concerns that IIOP episodes might simply be induced by subacute or intermittent angle-closure attacks, which also tend to be less painful and produce milder symptoms. We excluded this possibility because the IIOP attacks recurred even after patent laser iridotomies. After laser iridotomies, the gonioscopy exam revealed open angles and no evidence of iridotrabecular contact, except preexisting sporadic PAS in 4 cases (cases 1, 3, 4, and 9). Furthermore, by reviewing the charts, we found that a mydriatic agent was used for retina examinations in some patients, and no angle-closure attacks were recorded. Therefore, the plateau iris configuration is also not a likely cause of IIOP in these patients. We believe that the causes of these IIOP attacks are more closely related to their cyclic nature than angle features.

In our case series, the angle structure in individuals of Asian descent, presentation of the IIOP attacks, and findings on gonioscopy combined with the absence of typical symptoms of acute angle closure (severe pain, nausea, vomiting, and corneal edema) indicate that a variant of PSS may occur in patients with a narrow angle. Additionally, our patients tended to be older, which can explain the age-related lens effect on the decline in angle width. In contrast to typical PSS, PAS was present in some of our cases. However, further discussion may be needed to clarify the actual cause of this disease.

## 5. Conclusions

Based on our clinical observation, it is possible for patients with narrow or closed angles to have glaucomatocyclitic crises, which is different from the primary feature described by Posner and Schlossman. The variance of PSS and the concomitance between PSS and narrow angle/angle closure (suspect), especially in individuals of Asian descent, should be noted. PSS should not be excluded from the diagnosis of patients with acute IIOP episodes, shallow anterior chambers, white KP, patent laser iridotomies, and less severe symptoms, especially in Asians and middle-aged adults.

To the best of our knowledge, this case series is the first to present glaucomatocyclitic crisis episodes in individuals with a narrow angle/angle closure. We reported these cases to indicate the potential coexistence of narrow angle/angle closure, PAS, and glaucomatocyclitic crises.

## Figures and Tables

**Table 1:** Demographic data and a brief description of ocular examinations.

Cases	Age of PSS diagnosed/sex	Eye	Number of IIOP attacks	Highest IOP (mmHg)	Angle type (Shaffer system)	C/D ratio when PSS diagnosed	Visual field test after the first episode	Anterior chamber reaction grade on the first attack^†^	Subsequent long-term glaucoma medications use	Follow-up period (months)
1	51/female	Right	10	60	GrI~II with sporadic PAS	0.4	Normal	0.5+	Yes	443
2	53/female	Left	7	54	GrI~II	0.5	Normal	1+	Yes	193
3	64/male	Left	2	32	GrII with one PAS^‡^	0.45	Normal	1+	Yes	159
4	62/female	Right	2	58	GrI with 60° PAS	0.4	Normal	0.5+	No	8
5	46/female	Right	2	42	GrII	0.2	Not available	1+	No	3
6	52/male	Right	3	38	GrI~II	0.3	Normal	0.5+	No	33
7	75/female	Left	2	38	GrI~II^‡^	0.6	Normal	0.5+	No	76
8	62/male	Right	3	50	GrI	0.5	Normal	0.5+	No	96
9	54/female	Left	1	39	GrI with sporadic PAS^‡^	0.55	Normal	1+	No	18

PSS: Posner-Schlossman syndrome; IIOP: increased intraocular pressure; IOP: intraocular pressure; C/D ratio: cup to disc ratio; Gr: grade. ^†^The Standardization of Uveitis Nomenclature (SUN) Working Group Grading Scheme for Anterior Chamber Cells. ^‡^Angle recorded as a primary condition (before any laser iridotomy was performed).

**Table 2:** Primary description of glaucomatocyclitic crises by Posner and Schlossman [1, 3].

Descriptions	
(1)	Unilateral and recurrent
(2)	Typically neither posterior synechiae nor PAS
(3)	Mild discomfort or blurring of vision
(4)	Increased IOP with open angles
(5)	Mild anterior chamber reaction or fine white KP
(6)	Crises lasting from several hours to weeks
(7)	Normal IOP and no signs of uveitis between attacks
(8)	Normal visual fields and optic discs

PAS: peripheral anterior synechiae; KP: keratic precipitates.
